# Tropisetron Preconditioning Decreases Myocardial Biomarkers in Patients Undergoing Heart Valve Replacement Surgery

**DOI:** 10.3389/fmed.2022.690272

**Published:** 2022-03-29

**Authors:** Di Yu, Xingrui Gong, Yufei Zhang, Qing Li, Mazhang Zhang

**Affiliations:** ^1^Department of Anesthesiology, Taihe Hospital, Hubei University of Medicine, Shiyan, China; ^2^Hubei No. 3 People’s Hospital of Jianghan University, Wuhan, China; ^3^Department of Anesthesiology, Xiangyang Central Hospital, Hubei University of Arts and Science, Xiangyang, China; ^4^Department of Anesthesiology, Shanghai Children’ Medical Central, Shanghai Jiao Tong University School of Medicine, Shanghai, China

**Keywords:** tropisetron, heart surgery, myocardial injury, α7 nACh receptor, inflammation

## Abstract

**Background:**

Cardioplegic arrest during the heart valve replacement surgery frequently leads to myocardial damage. Tropisetron (TRP) has been demonstrated to reduce myocardial ischemia-reperfusion injury and inflammation in animals. We examined the efficacy of TRP in lowering myocardial biomarkers in patients undergoing heart valve replacement surgery.

**Methods:**

A total of seventy-five patients, scheduled for elective heart valve replacement surgery, were randomly chosen to receive either 10 ml of normal saline or 10 mg/10 ml of TRP immediately after anesthesia induction. Blood samples for the measurement of cardiac troponin I (cTnI), creatine kinase (CK-MB), lactate dehydrogenase (LDH), tumor necrosis factor-α (TNF-α), interleukin-1β (IL-1β), and interleukin-10 (IL-10) were taken before anesthesia, as well as 4, 12, and 24 h after aortic cross-clamp release to evaluate myocardial injury using two-way ANOVA for repeated measurements. The study was registered at www.chictr.org.cn (number, ChiCTR-1800018681).

**Results:**

Treatment with TRP decreased the increment of cTnI (*F*group = 4.911, *p* = 0.030; *F*time = 55.356, *p* = 0.001; *F*group × time = 5.340, *p* = 0.002) at 12 and 24 h; of CK-MB (*F*group = 6.552, *p* = 0.013; *F*time = 49.276, *p* = 0.001; *F*group × time = 7.627, *p* = 0.003) at 4, 12, and 24 h; of TNF-α (*F*group = 4.153, *p* = 0.046; *F*time = 28.244, *p* = 0.002; *F*group × time = 4.692, *p* = 0.006) at 4 and 12 h; and of LDH (*F*group = 4.275, *p* = 0.043; *F*time = 63.225, *p* = 0.001; *F*group × time = 2.501, *p* = 0.083) at 24 h after the release of the aortic cross-clamp. It increased IL-10 (*F*group = 5.958, *p* = 0.018; *F*time = 31.226, *p* = 0.002; *F*group × time = 1.464, *p* = 0.236) at 12 h after the release of the aortic cross-clamp. Multiple linear regression analysis showed that cardiopulmonary bypass (CPB) time was a risk factor, and that TRP treatment was a protective factor for postoperative cTNI change (β = 4.449, 95% CI [0.97–7.92], *p* = 0.013 for CPB time; and β = −381, 95% CI [−613.4 to −148.5], *p* = 0.002 for TRP treatment).

**Conclusions:**

Tropisetron had cardioprotective and anti-inflammatory effects in patients undergoing heart valve replacement surgery with cardioplegic arrest. The addition of TRP and reduction of CPB time should be considered for myocardial protection in heart valve replacement surgery.

**Clinical Trial Registration:**

[www.chictr.org.cn/index.aspx], identifier [ChiCTR1800018681].

## Introduction

Myocardial injury is one of the major factors for perioperative complications and mortality. During the heart surgery, ascending aorta cross-clamp and cardiac arrest result in myocardial ischemic damage (1). After cross-clamp release and subsequent oxygenated blood reperfusion, free oxygen radicals are released, which causes further myocardial injury. Importantly, ischemia-reperfusion (IR) injury and cardiopulmonary bypass (CPB) lead to a proinflammatory response and the release of various inflammatory cytokines such as interleukins, complement, histamine, serotonin, and other proinflammatory cytokines (2). Inflammation causes myocardial cell and vascular endothelial cell injury and ultrastructure damage, mitochondrial structure disorder, calcium overload and electrophysiological change, and even severe arrhythmia and death (3). Therefore, seeking effective measures to prevent perioperative inflammation is critical for perioperative myocardial protection and Prevention of Heart Injury-Related Complications.

Recent reviews have reported that 5-HT3 receptor antagonists have anti-inflammatory actions and are effective for treating neurologic and psychiatric disorders (4, 5). Tropisetron (TRP) is a frequently used drug for treating clinical nausea and vomiting by inhibition of 5-HT3 receptors. Moreover, serotonin, induced by surgery and CPB, activates 5-HT3 receptors and induces an inflammatory response, which aggravates myocardial injury after IR (6). Surprisingly, TRP has also been reported as a partial agonist of alpha7 nicotine acetylcholine (α7 n Ach) receptor. In addition, the activation of α7 n Ach receptor suppresses inflammation through the “cholinergic anti-inflammatory pathway” (7). Recently, studies on animals have demonstrated that TRP is cardioprotective due to its anti-inflammatory effect (8, 9). Thus, we designed this study to examine the cardioprotective effect of TRP in a clinical setting. We determined the efficacy of TRP in lowering perioperative cardiac biomarkers and inflammatory response in patients undergoing heart valve replacement surgery with cardioplegic arrest.

## Materials and Methods

The study was carried out in accordance with the Declaration of Helsinki (October 2000) outlined by the World Medical Association. The research protocol was approved by the institutional ethics committee of Taihe Hospital, Shiyan, China. After obtaining informed written consents from all subjects, a total of 75 patients aged 30–75 years, with an ASA physical status from 2 to 3, who were scheduled for mitral and/or aortic valve replacement, were scrutinized in this study. Exclusion criteria were as follows: patients with an LVEF <45%; any coronary artery disease with >70% stenosis; acute heart failure; myocardial infarction less than 4 weeks ago or angina within the previous week; uncontrolled hypertension; any brain, renal, pulmonary, or hepatic disease; any serious allergy to trial medications; any contraindication to midazolam, fentanyl, rocuronium, TRP, and propofol; and any administration of serotonin reuptake inhibitors in the previous 4 weeks.

Before the experiment, a biostatistician generated randomized numbers and allocated patients into two groups: group C, receiving 10 ml of normal saline; or group TRP receiving 10 mg/10 ml of TRP. After the admission to an operating room, a right internal jugular vein cannula and a radial arterial cannula were placed for hemodynamic monitoring under local anesthesia. Then, biostatistician prepared the drug according to the number sequence. An anesthesia assistant took the drug from the biostatistician and injected it using a pump within 20 min after anesthesia induction. For anesthesia induction, we used imidazoline (0.04 mg/kg), etomidate (0.2 mg/kg), and rocuronium (0.15 mg/kg), followed by sufentanil (1 μg/kg) administration. Then, 3 min later, endotracheal intubation was performed by an experienced anesthetist. Anesthesia in all of the patients was maintained with continuous infusions of propofol (50–150 μg/kg/min) and sevoflurane (1%). A total dose of sufentanil 5 μg/kg was used for analgesia during the surgery.

All of the patients were monitored with a standard 5-lead electrocardiograph. During the anesthesia, the mean arterial pressure was maintained within 20% from its baseline value and above 60 mm Hg, which was achieved either by infusion of 20 μg phenylephrine or by 20 μg nitroglycerin each time to increase or decrease the radial arterial pressure, respectively. If HR was above 90 or below 45 beats per min, 20 mg esmolol and 0.3 mg atropine were injected, and these treatments were repeated if necessary. After the release of the aortic cross-clamp, inotropic support with dopamine was considered if mean arterial pressure (MAP) was less than 60 mm Hg. In addition, nitroglycerin was infused continuously, and the infusion rate (0.1–10 μg/kg/min) changed depending on the central venous pressure (CVP) and MAP. CVP, MAP, and HR were recorded at end-expiration. The hemodynamic data at one time point were averaged from three repeated measurements with 2-min intervals while the patient was in a relatively stable hemodynamic condition.

The durations of CPB, operation, aortic occlusion, and intensive care unit (ICU) and postoperative hospitalization stay were recorded. Serial blood samples for the calculation of myocardial injury and inflammatory cytokines were taken before anesthesia and 4, 12, and 24 h after the release of the aortic clamp. The samples were quickly cooled down to 4°C and centrifuged at 4,000 rpm at 4°C for 5 min using cryogenic centrifuge (5840R, Eppendorf, Hamburg, Germany); then, the plasma samples were stored at −80°C for assay with ultralow-temperature freezer (TSE240V, Thermo Fisher Scientific, Waltham, MA, United States). cTnI, CK-MB, LDH, TNF-a, IL-1β, and IL-10 were measured using an ELISA kit in accordance with the instructions (RayBiotech, GA, United States for cTnI and CK-MB; Neobioscience Ltd., Shenzhen, China for TNF-a, IL-1β, and IL-10; Nanjing Jiancheng Bioengineering Institute, Nanjing, China for LDH). ELISA readings were done in line with the manufacturer’s procedures and read with an instrument (Multiskan FC, Thermo Fisher Scientific, Waltham, MA, United States). Abnormal levels of cTnI and CK-MB were defined as levels over 0.5 ng/ml and 0.6 ng/ml, respectively.

The sample size calculation used cTNI content with the formula: n1 = n2 = 2[(μα + μβ) × σ/δ]^2 + μα^2/4, based on a two-sided alpha error of 0.05 and power of 80%. From our preliminary data, the cTNI content was 538 ± 382 and 337 ± 133 pg/ml in the control and treatment groups, respectively (*n* = 12 per group), and *n* = 27 per group was required in each group in the experiment. Considering that 15–20% of patients were expected to possibly drop out from the experiment, we scrutinized 75 patients in total to evaluate the effect of TRP.

Categorical data were expressed by their number and analyzed using the chi-square or Fisher’s exact test. Quantitative data were expressed as means ± standard deviation (SD). Comparisons of quantitative data between the groups were done using the two-tailed Student’s *t*-test. Comparisons of biomarkers and hemodynamic data recorded over time between the groups were analyzed using two-way ANOVA for repeated measurements. If an overall significant difference between the groups was found, Bonferroni *post-hoc* tests were conducted. The statistical analyses were done using the GraphPad Prism software (GraphPad Prism 5.0, version 2.0; GraphPad Software Inc., San Diego, CA, United States 5.0). The study was registered at www.chictr.org.cn with the number ChiCTR-1800018681.

## Results

### Patients’ Baseline Characteristics and Perioperative Hemodynamics

**FIGURE 1:**
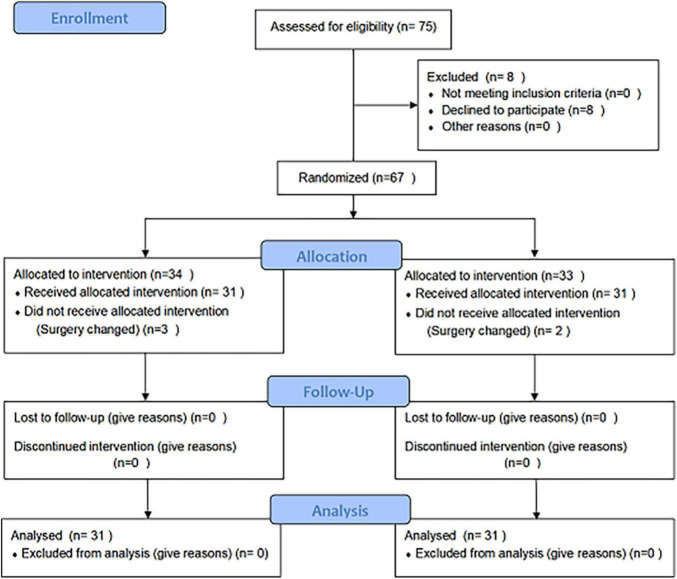
Trial flowchart.

Out of a total of 75 patients who were scrutinized, eight patients refused to participate in the experiment. A number of three and two patients in the control and TRP group, respectively, were excluded due to changes in the surgery according to the assessment of the surgeon during the surgery. Ultimately, 62 patients met the criteria and were included in our study, and all of the surgeries were successfully conducted by the same surgical team. Two patients died in the control group (7 and 19 days after surgery) because of heart failure (Figure 1).

Patients’ characteristics in the two groups were comparable in terms of sex, preexisting medical conditions, and comorbidities (*p* > 0.05, *t*-test or chi-square test, Table 1). The anesthesia and surgery time, as well as the duration of hospital stay, were not different between the groups (*p* > 0.05, *t*-test, Table 2). The duration of ICU stay in the TRP group was shorter than that in the control group (*p* < 0.05, *t*-test, Table 2). The HR, MAP, and CVP were not different before the surgery or 12 and 24 h after surgery between the TRP and the control groups (*p* > 0.05, two-way ANOVA for repeated measurements, Table 3).

**TABLE 1:** Patients baseline characteristics.

Characteristic	Control group (*n* = 31)	TRP group (*n* = 31)	*P*-value
Age (years)	50.5 ± 11.4	48.7 ± 10.8	0.517
Sex ratio (M/W)	21/10	17/14	0.297
Height (cm)	165.5 ± 6.3	163.8 ± 6.6	0.330
Weight (kg)	61.0 ± 10.8	61.2 ± 11.6	0.648
BMI (kg/m^2^)	22.2 ± 2.9	22.6 ± 2.9	0.522
EF (%)	59.7 ± 5.9	56.8 ± 6.6	0.072
LEVDd (mm)	51.7 ± 9.2	49.8 ± 8.9	0.418
LAD (mm)	47.5 ± 12.9	45.8 ± 10.7	0.593
Cardiac function (NYHA)			
II, n (%)	7 (22.6)	9 (29.0)	0.562
III, n (%)	24 (77.4)	22 (71.0)	0.562
Type of surgery, n (%)			
Mitral valve replacement	13 (41.9)	15 (48.4)	0.610
Aortic valve replacement	12 (38.7)	10 (32.3)	0.596
Joint valve replacement	6 (19.4)	6 (19.4)	–
Hypertension, n (%)	6 (19.4)	10 (32.3)	0.246
Coronary artery disease, n (%)	6 (19.4)	5 (16.1)	0.740
Rheumatic heart disease, n (%)	25 (80.6)	29 (93.5)	0.256

*Results are expressed as mean ± SD. M/W, men/women; BMI, body mass index; EF, ejection fraction; LEVDd, left ventricular end-diastolic dimension; LAD, left atrial diameter; NYHA, New York Heart Association.*

**TABLE 2:** Patients’ perioperative variables.

Variables	Control group (*n* = 31)	TRP group (*n* = 31)	*P*-value
CPB time (min)	104.3 ± 27.7	109.2 ± 39.2	0.566
Aortic cross-clamp time (min)	71.8 ± 32.0	68.2 ± 31.1	0.659
Operation time (min)	251.9 ± 55.0	251.5 ± 68.5	0.976
Duration of ventilation (h)	35.8 ± 45.6	29.8 ± 34.5	0.559
Length of ICU stay (h)	85.8 ± 68.8	52.1 ± 40.1*	0.022
Postoperative hospital stay (days)	19.7 ± 7.8	18.97 ± 6.0	0.690

*Results are expressed as mean ± SD.*

*P < 0.05 compared with the control group.

*SD, standard deviation; CPB, cardiopulmonary bypass; ICU, Intensive care unit.*

**TABLE 3:** Patients’ perioperative hemodynamic data.

Time	Heart rate		MAP		CVP	
	Control group	TRP group	Control group	TRP group	Control group	TRP group
T0	86.5 ± 24.4	84.8 ± 15.1	89.9 ± 14.0	90.3 ± 8.9	7.3 ± 2.2	7.4 ± 2.1
T1	95.2 ± 18.8	91.5 ± 14.0	77.1 ± 9.5	77.5 ± 5.7	7.9 ± 1.9	7.5 ± 1.9
T2	99.8 ± 13.7	96.7 ± 13.2	77.9 ± 10.8	78.4 ± 9.2	7.7 ± 2.5	7.6 ± 2.4
T3	97.8 ± 7.4	98.6 ± 10.8	79.0 ± 8.0	81.35 ± 7.3	8.2 ± 2.0	8.1 ± 2.3
T4	98.2 ± 9.8	100.0 ± 7.7	80.4 ± 11.4	82.3 ± 10.8	10.5 ± 2.4	8.9 ± 2.5
T5	99.4 ± 8.4	98.7 ± 10.1	80.95 ± 7.1	83.4 ± 13.4	10.3 ± 2.3	9.4 ± 2.7

*Results are expressed as mean ± SD. SD, standard deviation; MAP, mean arterial pressure; CVP, central venous pressure.*

Routine blood tests showed that white blood cell (WBC), neutrophil (NE), and platelet (PLT) counts were not different between the control and the TRP groups. However, CRP was lower in the TRP group than in the control group 24 h after the cross-clamp release (*p* < 0.05, *t*-test, Table 4). None of the patients were diagnosed with acute myocardial infarction, stroke, hepatic, or renal failure during the hospital stay. Postoperative complications, which include ventricular arrhythmia, perioperative myocardial infarction, cerebrovascular accidents, abnormal coagulation function, infection, stroke, hospital death, and endocarditis, were not different between the control and TRP groups (*p* > 0.05, chi-square test, Table 5).

**TABLE 4:** Routine blood tests before and 24 h after heart surgery.

Time		Control group (*n* = 31)	TRP group (*n* = 31)	*P*-value
Before	WBC	6.4 ± 1.9	6.9 ± 2.6	0.407
	NE	4.1 ± 1.5	4.8 ± 2.8	0.249
	PLT	199.9 ± 51.9	185.5 ± 47.3	0.257
98QJ	CRP	1.7 ± 2.1	1.8 ± 1.8	0.742
After	WBC	16.3 ± 4.1	16.3 ± 4.8	0.964
	NE	14.4 ± 4.2	15.0 ± 4.6	0.647
	PLT	136.4 ± 42.1	134.2 ± 36.6	0.828
	CRP	149.8 ± 70.8	120.8 ± 33.7*	0.043

Results are expressed as mean ± SD. *P < 0.05 compared with the control group. SD, standard deviation; WBC, white blood cell; NE, neutrophil; PLT, platelet; CRP, C-reactive protein.

**TABLE 5:** Postoperative complications.

Complications	Control group (*n* = 31)	TRP group (*n* = 31)	*P*-value
Ventricular arrhythmia, n (%)	7 (22.6)	5 (16.1)	0.520
Perioperative MI, n (%)	0	0	–
Cerebrovascular accidents, n (%)	0	0	–
Abnormal coagulation function, n (%)	4 (12.9)	2 (6.5)	0.668
pulmonary Infection, n (%)	6 (19.4)	4 (12.9)	0.490
Shock, n (%)	1 (3.2)	0	1.000
Endocarditis, n (%)	0	0	–
Hospital death, n (%)	2 (6.5)	0	0.472

### Changes in the Biomarkers of Myocardial Injury

**FIGURE 2:**
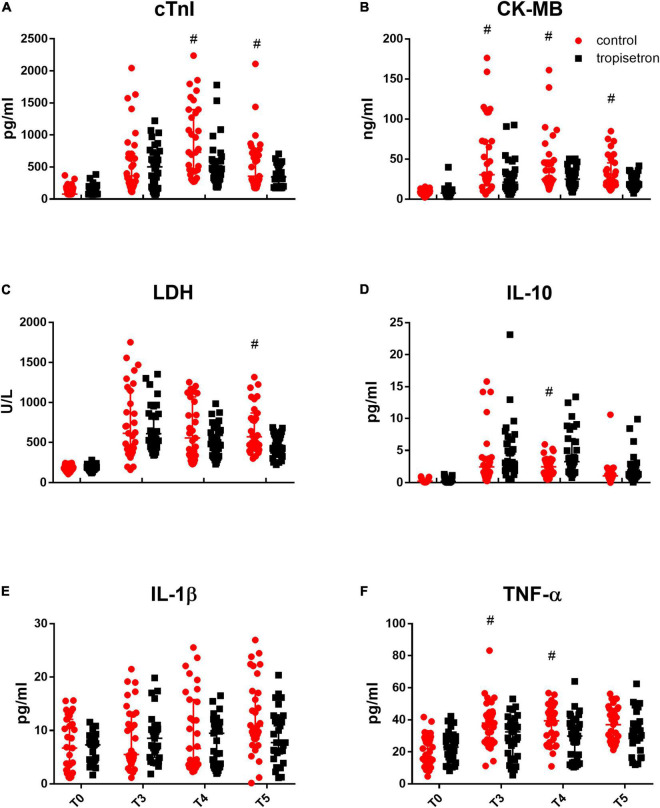
Effect of TRP on perioperative myocardial biomarkers and inflammatory cytokines. Myocardial injury biomarkers, which include **(A)** cTnI, **(B)** CK-MB, and **(C)** LDH, increased after the release of aortic cross-clamp, while TRP treatment decreased that effect (*p* < 0.05, two-way ANOVA for repeated measurements followed by Bonferroni *post-hoc* tests). Inflammatory cytokines, such as **(D)** IL-10, **(E)** IL-1β, and **(F)** TNF-α, were increased, while TRP treatment increased IL-10 and decreased TNF-α levels compared with the control group after the release of the aortic cross-clamp (*p* < 0.05, two-way ANOVA for repeated measurements followed by Bonferroni *post-hoc* tests). ^#^*P* < 0.05 compared with the control group.

Myocardial injury biomarkers (cTnI, CK-MB, and LDH) were measured, and the results showed that cTnI, CK-MB, and LDH (Figures 2A–C) were not different between the groups before anesthesia. Their values increased at 4 h after the aortic clamp release, with CK-MB and LDH peaking at 4 h and cTnI peaking at 12 h, compared to the baseline values (*p* < 0.05, two-way ANOVA for repeated measurements followed by Bonferroni *post-hoc* tests; Figures 2A–C). In addition, the administration of TRP after anesthesia decreased the levels of cTnI at 12 and 24 h; of CK-MB at 4, 12, and 24 h; and of LDH at 24 h after the aortic clamp release.

### Changes in Inflammatory Cytokines

The cytokine contents of IL-10, TNF-α, and IL-1β in the serum were not different between the groups before anesthesia and were increased after the aortic clamp release when compared to the baseline values. The addition of TRP after anesthesia increased the level of IL-10 at 12 h and decreased the level of TNF-α at 4 and 12 h after the aortic clamp release (*p* < 0.05, two-way ANOVA for repeated measurements followed by Bonferroni *post-hoc* tests, Figures 2D–F), whereas it did not affect IL-1β content.

### Correlation and Multivariate Regression Analysis of Potential Risk Factors for Myocardial Injury

Linear correlation analysis was conducted to explore the potential association of cTNI with age, gender, BMI, CPB time, WBC, NE, PLT, CRP, EF, LVEDD, LAD, and TRP treatment (Table 6). The results showed that cTNI correlated with CPB time, CRP level, and TRP treatment (r = 0.268, *p* = 0.035 for CPB time; r = 0.024, *p* = 0.06 for CRP; r = −0.357, *p* = 0.004 for TRP treatment). Then, CPB time, CRP level, and TRP treatment were included in the multivariate regression analysis. The results showed that CPB time was a risk factor, whereas TRP treatment was a protective factor for postoperative cTNI change (β = 4.449, 95% CI [0.97–7.92], *p* = 0.013 for CPB time, and β = −381, 95% CI [−613.4 to −148.5], *p* = 0.002 for TRP treatment).

**TABLE 6:** Correlation and multivariate regression analysis of potential risk factors for cTnI 12 h after surgery.

Independent variable	Correlation analysis		Multivariate linearity		
			Regression analysis		
	r	p	β	95%CI	p
Age	–0.097	0.455			
Gender	0.103	0.424			
BMI	–0.059	0.647			
CPB time	0.268	0.035	4.449	0.97–7.92	0.013
WBC	–0.123	0.342			
NE	–0.061	0.637			
PLT	–0.062	0.634			
CRP	–0.024	0.060	−52.183	−112.18 to 7.82	0.087
EF	0.034	0.791			
LEVDd	0.135	0.294			
LAD	–0.172	0.181			
Treatment	–0.357	0.004	−380.998	−613.4 to −148.5	0.002

*BMI, Body mass index; CPB, cardiopulmonary bypass; WBC, white blood cell; NE, neutrophil; PLT, platelet; CRP, C-reactive protein; LEVDd, left ventricular end-diastolic dimension; LAD, left atrial diameter.*

## Discussion

The results of this study suggested that TRP provides superior myocardial protection over normal saline in patients undergoing heart valve replacement surgery. The conclusions were verified by lower concentrations of cTnI, CK-MB, and LDH in the TRP group after surgery.

In our experiment, the surgery of heart valve replacement involved aortic cross-clamp, cardiac arrest, and reperfusion. Since IR frequently contributes to myocardial injury and biochemical marker elevation, we chose patients scheduled for valvular replacement surgery as our target population to evaluate the cardioprotective effects of TRP. In this study, we measured myocardial injury, which includes cTnI, CK-MB, and LDH. Furthermore, cTnI is a conventional myocardial contractile protein, which can be detected in the blood 3–6 h after the cessation of regional coronary blood flow. It is a reliable marker for evaluating myocardial damage and is more sensitive and specific than other serum enzymes. The study has recommended cTnI for the early diagnosis of acute coronary syndromes, and cTnI also has a good prognostic value (10). Creatine kinase (CK) has three cytoplasmic isozymes in three kinds of tissues, which include CK-MB (heart), CK-MM (muscle), and CK-BB (brain). After the onset of coronary syndrome, the concentration of CK-MB begins to increase within approximately 4–6 h, peak after 17 h (±1 h), with a half-life of 11 h (±1 h) in plasma (11). In clinical medicine, CK-MB plays an essential role as a biomarker in the diagnosis and the assessment of myocardial infarction in combination with cTnI. LDH is a traditional enzyme that catalyzes the pyruvic acid to lactic acid. In the setting of myocardial damage, LDH is released into serum; it has been verified for the assessment of myocardial injury, as it correlates well with the degree of injury (12). These markers increase and peak immediately after the myocardial injury. Since the first 24 h is the most critical for the development of myocardial accident, these three biochemical markers were chosen and tested before anesthesia, as well as at 4, 12, and 24 h after the cross-clamp release, to evaluate myocardial injury.

Our results showed that the levels of cTnI, CK-MB, and LDH were increased after the release of the aortic cross-clamp, but TRP decreased those increments, which suggests a cardioprotective effect of TRP. A previous study has demonstrated the cardioprotective effects of TRP both *in vivo* and *in vitro*. Doxorubicin has a life-threatening cardiotoxic effect in animals. However, a prior intraperitoneal injection of TRP has been shown to improve heart contractility and electrocardiographic changes in addition to decreasing the mortality rate induced by doxorubicin in animal experiments. In addition, TRP robustly counteracted the increment in serum biomarkers and alleviated the histopathological changes compared with the control group (8). Trauma-hemorrhage shock results in excessive production of proinflammatory mediators, such as cytokines and chemokines. Multiple organ failure or dysfunction secondary to a systemic inflammatory response is a major cause of mortality and morbidity. Nevertheless, the administration of TRP significantly improves multiple organ dysfunction, cardiac function, and survival (9). The mechanisms may involve the prevention of inflammation and apoptosis of cardiac cells in rats subjected to hemorrhage shock. Furthermore, an *in vitro* study has shown that TRP may have protective effects against high glucose-induced cardiomyocyte hypertrophy. The mechanism responsible for this beneficial effect seems to be, at least in part, due to a blockade of calcineurin–nuclear factor of an activated T-cell signaling pathway (13). Generally, consistent with the previous preclinical studies, our clinical outcomes suggested that TRP had a cardioprotective effect after CPB.

Such a cardioprotective effect may be attributed to the activation of α7 n Ach receptors and the inhibition of 5-HT3 receptors. Recently, α7 n Ach receptor activation has been demonstrated to present cardioprotective effects. According to this report, activating α7 n Ach receptors at the initiator stage of reperfusion reduces myocardial infarct size by inhibiting Beclin-1 and cascading of signaling pathways against IR injury (14). In addition, high-sensitivity CRP increased IL-6 level, p38MAPK expression, and monocyte activation, which contribute to coronary artery spasm, whereas the overexpression of the monocytic α7 n Ach receptor decreases oxidative stress and inflammation-associated coronary artery spasm (15). Inflammation is a major cause of myocardial IR injury, while vagal nerve stimulation presents an anti-inflammatory effect and reduces the infarct size through α7 n Ach receptor activation (16, 17). Importantly, Nrf2 is a transcriptional factor and has a pivotal role in redox signaling; TRP activates Nrf2 *via* α7 n AChR, which results in apoptosis inhibition (18). Also, TRP exerts notable anti-inflammatory effects through peroxisome proliferator-activated receptor gamma, another crucial transcriptional factor that regulates anti-inflammatory signaling (19). Generally, TRP presents anti-inflammatory effects by the activation of α7 nAch receptors. Unlike other anti-inflammatory medications, α7 n Ach receptor activation is a part of the inner “cholinergic anti-inflammatory pathway”; thus, it has minimum side effects. However, the downstream mechanisms of α7 n Ach receptor activation are still very complex and poorly understood.

In addition, 5-HT3 receptor inhibition may contribute to TRP-induced myocardial protection. Surgery, trauma, CPB surgery, and cardiac arrest increase the release of serotonin and activation of 5-HT3 receptors. 5-HT3 receptor activation mediates platelet activation and thrombosis after cardiac ischemic damage, and inhibition of 5-HT3 receptors prevents platelet activation and thrombosis and reduces ischemic damage after valve replacement surgery (20). Moreover, serotonin activates cardiac sympathetic afferents through the stimulation of 5-HT3 receptors, which results in elevated plasma levels of norepinephrine and cardiac dysfunction. Inhibition of 5-HT3 receptors reduces the elevated plasma level of norepinephrine in mice, prevents cardiac hypertrophy, and restores desensitization of cardiac β-adrenergic receptors in aortic banding-treated rats (21). Sepsis is a severe infection that aggravates myocardial structural changes because of proinflammatory cytokines; however, the addition of a 5-HT3 receptor antagonist significantly inhibits the cytokines’ overexpression and myocardial injury in sepsis (22). 5-HT3 receptor agonists cause a rapid depolarization of the membrane potential, which results in the opening of cation channels and Ca^2+^ inflow, while the blockade of 5-HT3 receptors diminishes intracellular Ca^2+^ overload and decreases reactive oxygen species and glutamate excitotoxicity (23). Thus, 5-HT3 receptor activation-related inflammation suppression is another critical factor for the cardioprotective effect of TRP. The effects are mainly mediated by platelets inhibition, sympathetic afferent suppression, and cation channels blockade.

Inflammation is an independent risk factor for the development of myocardial injury after cardiac stunning (24). In our study, we choose IL-10, TNF-α, and IL-1β as the inflammatory biomarkers for evaluating the anti-inflammatory effects of TRP. Our results showed that TRP decreased the increment of TNF-α postoperatively. TNF-α has been demonstrated to participate in myocardial IR by promoting leukocyte infiltration of the myocardium, while TNF-α knockout mice have decreased arrhythmia and improved cardiac function during reperfusion (25). TNF-α also induces long-term cardiac contractile dysfunction, hypertrophy, fibrosis, and cell death (26). The expression of chemokines and adhesion molecules and the infiltration of leukocytes were significantly reduced in TNF-α knockout mice. IL-1β is critically involved in the postinfarction inflammatory reaction, and it mediates adverse dilative remodeling (27). In contrast, IL-1β inhibition improves adverse cardiac remodeling after acute myocardial infarction, which includes left ventricle end-systolic volume index, change in CRP levels, and proinflammatory response (28). However, our results did not show a significant difference in IL-1β levels between the two groups. IL-10 has been demonstrated to improve cardiac remodeling after myocardial infarction by stimulating M2 macrophage polarization and fibroblast activation (29). The mechanism for the cardioprotective effect of IL-10 may involve the IL-10–STAT3–galectin-3 axis (30). In our experiment, IL-10 was increased and TNF-α was decreased after TRP treatment, which suggests that the cardioprotective effects of TRP may be attributed to the overexpression of IL-10 and suppression of TNF-α expression.

In addition to the activation of α7 n Ach receptors and the inhibition of 5-HT3 receptors, TRP may confer cardioprotective effect through other mechanisms. MAPK is an acute proinflammatory protein; it is activated after injury and mediates the proinflammatory response. TRP has been demonstrated to reverse lipopolysaccharide-induced TNF-α and IL-1β expression *via* inhibition of p38 MAPK activation in the monocyte (31). Activation of the 5-HT3 receptor allows Ca^2+^ entry, which results in Ca^2+^ overload and aggravates cardiac injury (6). TRP has also been shown to block cation channels, which include cardiac potassium and sodium currents and exhibit mixed class III and class I antiarrhythmic properties in ventricular myocytes after ischemia (32, 33). These effects result in reduced myocardial oxygen consumption and myocardial protection. In addition to the cardiovascular effect, TRP presents immunoregulatory effects in stroke (34) and autoimmune diseases, which includes multiple sclerosis and experimental autoimmune encephalomyelitis (34). TRP decreases the size of infarct volume and inflammatory cytokine release induced by an autologous clot into the middle cerebral artery. TRP also decreases TNF-α, COX-2, iNOS, NF-KappaB, active caspase 3, cytochrome c release, and calcineurin phosphatase activity induced by Aβ neurotoxicity in the hippocampus (35). Importantly, it has been shown that TRP is a potent inhibitor of calcineurin, a canonical enzyme that regulates immune responses (36). Thus, in our study, the cardioprotective effect of TRP may be attributed to multiple anti-inflammatory pathways.

Furthermore, we used several anesthetics, including sufentanil (37) and propofol (38), which have been shown to provide myocardial protective effects. Since it was mandatory procedure to use these anesthetics, this study minimized the bias using the same dosage which was calculated according to the patients’ body weight. In both of the groups, sufentanil 5 μg/kg was used for each patient. Propofol 50–150 μg/kg/min was used continuously in both groups during the surgery. The effects of sufentanil and propofol were equal between the two groups perioperatively. Thus, the TRP-related decrease in myocardial biomarkers could not be attributed to sufentanil and propofol.

Our results showed that the baseline characteristics, which include age, gender, weight, LVEF, and preoperative medications, were all comparable between the two groups, which excluded the baseline imbalance. The surgery and anesthesia time and perioperative HR, BP, and CVP were not different between the groups. In addition, all of the surgeries were performed by the same surgery and anesthesia groups, which minimized the bias between the two groups. The cross-clamp time in the TRP group was less than that in the control group, which may have compromised the conclusions. There are several limitations to this study. It was a small population clinical trial, so the results are not suitable for assessing the long-term survival rate and the incidence of adverse outcomes, which includes myocardial infarction, stroke, and death. Furthermore, the anesthesia depth was not measured; however, we used the same anesthesia regime for all patients to minimize the bias.

In conclusion, our study showed that TRP preconditioning reduced myocardial biochemical markers and proinflammatory responses in patients undergoing heart valve replacement surgery with cardioplegic arrest. TRP preconditioning is a safe and effective approach for myocardial protection in patients with cardiovascular disease.

## Data Availability Statement

The raw data supporting the conclusions of this article will be made available by the authors, without undue reservation.

## Ethics Statement

The studies involving human participants were reviewed and approved by the Institutional Ethics Committee of Taihe Hospital, Shiyan, China. The patients/participants provided their written informed consent to participate in this study.

## Author Contributions

DY, XG, and YZ performed the experiment. XG, QL, and MZ designed the experiment. All authors contributed to the article and approved the submitted version.

## Conflict of Interest

The authors declare that the research was conducted in the absence of any commercial or financial relationships that could be construed as a potential conflict of interest.

## Publisher’s Note

All claims expressed in this article are solely those of the authors and do not necessarily represent those of their affiliated organizations, or those of the publisher, the editors and the reviewers. Any product that may be evaluated in this article, or claim that may be made by its manufacturer, is not guaranteed or endorsed by the publisher.

## Abbreviations

α7 n Ach: Alpha7 nicotinic acetylcholine
5-HT: 5-hydroxytryptamine
BP: blood pressure
CPB: cardiopulmonary bypass
CK-MB: creatine kinase isoenzyme
cTNI: cardiac troponin I
C VP: central venous pressure
HR: heart rate,
IL-1β: interleukin-1β
IL-10: interleukin-10
LVEF: left ventricular ejection fraction
LDH: lactate dehydrogenase
MAP: mean arterial pressure
MAPK: mitogen-activated protein kinase
TNF-α: tumor necrosis factor-α
TRP: tropisetron.
